# Genetic Overlap Between Type 2 Diabetes and Depression in a Sri Lankan Population Twin Sample

**DOI:** 10.1097/PSY.0000000000000771

**Published:** 2020-02-07

**Authors:** Carol Kan, Kaushalya Jayaweera, Anushka Adikari, Sisira Siribaddana, Helena M.S. Zavos, Lisa Harber-Aschan, Athula Sumathipala, Matthew Hotopf, Khalida Ismail, Frühling Rijsdijk

**Affiliations:** From the Department of Psychological Medicine, Institute of Psychiatry, Psychology and Neuroscience (Kan, Hotopf, Ismail), King’s College London, London, United Kingdom; Institute for Research and Development (Jayaweera, Adikari, Sumathipala), Colombo; Department of Medicine, University of Rajarata (Siribaddana), Mihintale, Sri Lanka; Department of Psychology, Institute of Psychiatry, Psychology and Neuroscience (Zavos), King’s College London, London, United Kingdom; Department of Public Health Sciences (Aschan), Karolinska Institutet, Solna, Sweden; Research Institute for Primary Care and Health Science (Sumathipala), Keele University, Newcastle, United Kingdom; and Social Genetic and Developmental Research Centre, Institute of Psychiatry, Psychology and Neuroscience (Rijsdijk), King’s College London, London, United Kingdom.

**Keywords:** type 2 diabetes, depression, genetic, twin, structural equation modeling, **AIC** = Akaike information criterion, **HbA_1c_** = glycated hemoglobin, **GWAS** = genome-wide association studies, **T2DM** = type 2 diabetes

## Abstract

**Objective:**

Results from twin studies examining the genetic overlap between type 2 diabetes and depression are currently inconclusive. This question has not been addressed in non-Western populations. We aimed to examine whether there are common genetic factors between type 2 diabetes and depression in a Sri Lankan population using genetic model-fitting analysis.

**Method:**

The Colombo Twin and Singleton Study–Phase 2 consists of 2019 singletons, and 842 monozygotic and 578 dizygotic twin pairs. The primary outcomes were self-reported type 2 diabetes diagnosis and Beck Depression Inventory scores. Standard bivariate twin models were fitted to estimate the genetic and environmental (co)variance of type 2 diabetes and depression.

**Results:**

In the best-fitting model, the phenotypic correlation between type 2 diabetes and depression was significant in female individuals only (*r* = 0.15 [0.08–0.21]). This association was primarily attributed to a significant genetic correlation between the traits (rA = 0.53 [0.19–0.98]).

**Conclusions:**

In female individuals, but not male individuals, we found a significant genetic overlap between type 2 diabetes and depression in the context of a modest phenotypic correlation.

## INTRODUCTION

Type 2 diabetes (T2DM) and depression are common disorders with considerable impact at personal, societal, and national levels. An association between T2DM and depression is well documented in epidemiological studies, with up to 60% increased risk for developing T2DM in individuals with depression and 15% for incident depression in those with T2DM alone (1,2). Depression is significantly associated with suboptimal glycemic control, higher complication rates, and increased mortality in people with T2DM (3–5). In addition, systemic inflammation, hypercortisolism, and disturbed immune functions have been demonstrated to contribute to the T2DM-depression association (6–8). Similar neuroimaging changes in white and gray matters have been observed in both people with T2DM and depression (9). There is now increasing evidence for common biological mechanisms being at play in the causal pathway for both T2DM and depression (10). It is therefore plausible that genetic pleiotropy between T2DM and depression might also explain some of the comorbidity observed. Furthering our understanding of the underlying mechanism of the T2DM-depression association will allow us to develop treatments and improve outcomes in this high-risk comorbid group.

Four twin studies have examined the genetic overlap between T2DM and depression. Two studies reported no evidence of correlated genetic factors (11,12), whereas qualitative and quantitative sex differences were reported in the (genetic) association of T2DM and depression in two large Scandinavian populations (13). Two studies using a polygenic score approach (14,15) and two studies using a linkage disequilibrium score approach (16,17) in genome-wide association studies (GWAS) have, however, reported no evidence of a genetic overlap between T2DM and depression. These studies were conducted in Western populations. The only non-Western population study examining this association from a genetic perspective was conducted collectively in six ethnic groups, namely, East Asian, South Asian, European, African, Latin American, and Native North American (14). Although the association between T2DM and depression has been observed in non-Western populations (18–20), its genetic determinants have yet to be examined and there are reasons to suggest that these might be different. In addition, the point prevalence of depression has been reported to vary with the human development index (21), whereas the prevalence of T2DM in non-Western populations is rapidly increasing, with a younger age of onset and greater mortality, in comparison with Western populations (22). Furthermore, previous twin studies have raised the possibility that the genetic architecture of depression might be different in non-Western populations, especially in male individuals (23). To further complicate the picture, epidemiological studies in Western populations have reported a substantially higher prevalence rate of comorbid T2DM and depression in female compared with male individuals (24–26). A genetic model incorporating possible quantitative and qualitative sex differences in genetic and environmental effects is therefore called for. In this study, we aimed to examine the genetic overlap of T2DM and depression in a South-Asian (Sri Lankan) twin population sample using sex-limitation genetic model-fitting analysis.

## METHODS

### Sample

The Colombo Twin and Singleton Study (CoTaSS) is a population-based sample of twins and a comparable sample of nontwins (singletons) born in the Colombo district of Sri Lanka, with >90% participation rate (23). CoTaSS-2 is a follow-up of the original study and was conducted between 2012 and 2014, with a >75% participation rate (83% in twins, 62% singletons) (27). In brief, CoTaSS-2 was designed to examine the relationship between metabolic risk factors and mental health. Written informed consent was obtained from all participants. Demographic and phenotypic data were collected through extensive health care questionnaires, whereas anthropometric and biological data were collected by trained research assistants. The study received ethical approval from the Psychiatry, Nursing and Midwifery Research Ethics Subcommittee at King’s College London (United Kingdom; reference number: PNM/10/11-124) and the Faculty of Medical Sciences Ethical Review Committee at the University of Sri Jayewardenepura (Sri Lanka; reference number: 596/11).

### Outcome Variables

T2DM was defined as the presence of T2DM as reported by the participants. In addition, fasting blood glucose and glycated hemoglobin (HbA_1c_) levels were collected 8 months after participants were recruited into the study. Depression was measured using the Beck Depression Inventory (BDI), which captures depressive symptoms and severity in the past 2 weeks (28). The BDI was translated into Sinhalese by a panel of clinical professionals fluent in both Sinhalese and English. The BDI questionnaire was cross-culturally adapted in wording to best describe the questions in their meaning (29) and has been previously been validated in the Sri Lankan population (23). Secondary variables included self-reported age and sex. Zygosity of same-sex twin pairs was based on a standard self-report questionnaire measure of similarity (30).

### Statistical Analysis

The classical twin method builds on the following three main assumptions: (i) monozygotic (MZ) twins share 100% and dizygotic (DZ) twins share on average 50% of their segregating genes (additive genetic effects); (ii) MZ and DZ twins are correlated for environmental influences to the same extent (equal environment assumption); and (iii) mating in the population occurs at random (nonassortative mating). In a univariate ACE model, individual differences in a trait are assumed to arise from additive genetic (A), common environmental (C), and unique environmental (E) influences. In a bivariate ACE model, in addition to the A, C, and E components of each trait, the phenotypic correlation between two traits can be partitioned into correlating addictive genetic (rA), shared environmental (rC), and unique environmental (rE) effects (31). Having same-sex male and female MZ and DZ twin pairs as well as opposite-sex twin pairs allows for testing for (i) “qualitative sex differences” where different genetic and common environmental factors are involved in male and female individuals, and (ii) “quantitative sex differences” where the same genetic and environmental factors are involved, but the magnitude of their effect is modulated by sex. The power to estimate qualitative sex differences is based on differences of within-trait and cross-trait correlations in opposite-sex DZ pairs compared with same-sex DZ pairs, whereas the power to estimate quantitative sex differences is based on differential MZ and DZ within-trait and cross-trait correlations in same-sex twin pairs.

A full sex-limitation model was first fitted in which the A, C, and E parameters were allowed to differ between male and female individuals, allowing to test for quantitative sex differences. In addition, for opposite-sex pairs, the correlations between the A factors and the C factors between male and female individuals were estimated freely in succession. These two models were then compared with the model in which the correlations between the A factors were constrained to 0.5 and those between the C factors to 1 in opposite-sex pairs, respectively. This allows us to test for qualitative sex differences. Equating the male and female parameters allows us to test for quantitative sex differences. The software program OpenMx (32) was used for genetic model-fitting analysis on combined dichotomous T2DM data and continuous, log-transformed sex- and age-regressed BDI residual scores. Age effects on T2DM were modeled on the liability threshold.

Two criteria were used to choose the best-fitting model: (i) differences in minus twice the log likelihood (−2LL) distributed as χ^2^ and (ii) Akaike information criterion (AIC) (33), with lower values indicating a better balance between explanatory power and parsimony and a difference in AIC of ≥10 indicating substantial support in favor of the more parsimonious model.

## RESULTS

### Descriptive Statistics

The CoTASS-2 sample used in this analysis consisted of 3956 twin individuals (1963 twin pairs and 30 twin individuals) and 2019 singletons. Of the twin individuals, 42.8% was MZ, 29.8% was same-sex DZ, and 27.4% was opposite-sex DZ (Table 1). The mean (SD) age was 43.0 (14.3) years, and mean (SD) body mass index was 23.8 (4.6) kg/m^2^. There were 471 cases with self-reported T2DM in total. For the entire sample, the mean (SD) fasting plasma glucose was 6.0 (2.3) mmol/L. The mean (SD) HbA_1c_ was 42.1 (15.3) mmol/mol as per International Federation of Clinical Chemistry (IFCC) units and 6.0% (1.4%) as per Diabetes Control and Complications Trial (DCTT) units. The intra-assay and interassay coefficients of variations for HbA_1c_ were 0.6 and 2.66 from set 1 and 0.32 and 2.61 from set 2, respectively. For individuals with a self-reported diagnosis of T2DM, the mean (SD) fasting plasma glucose was 8.9 (4.3) mmol, and mean (SD) HbA_1c_ values were 3.8 (47.0) mmol/mol) as per IFCC and 8.9% (4.3%) as per DCTT. For individuals who did not report a diagnosis of T2DM, the mean (SD) fasting plasma glucose was 5.6 (1.4) mmol/L, and mean HbA_1c_ was 38.8 (9.8) mmol/mol as per IFCC and 5.7% (0.9%) as per DCTT.

**TABLE 1:**
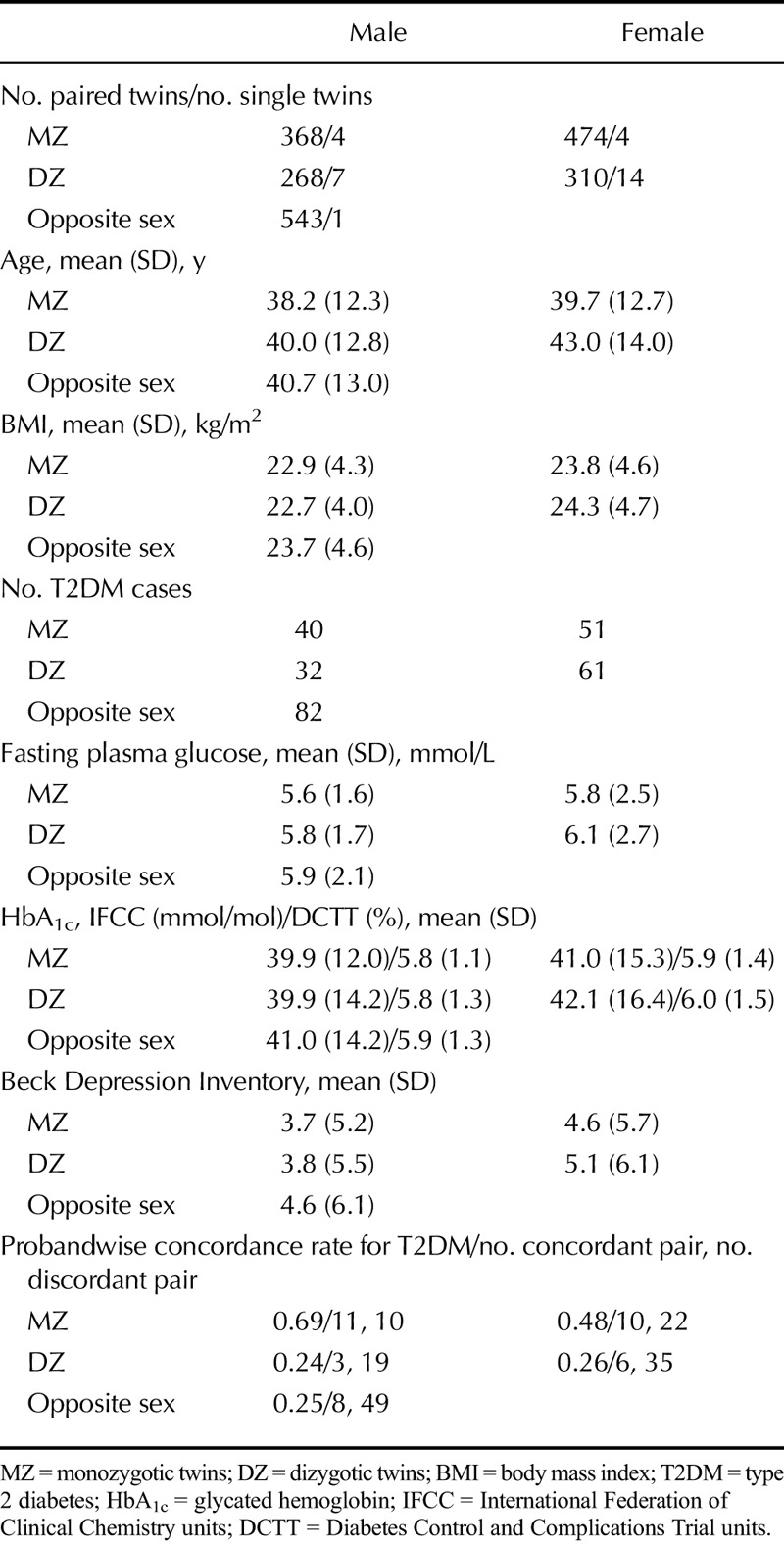
Descriptive Statistics of Monozygotic and Dizygotic Twins, Stratified by Sex

**FIGURE 1:**
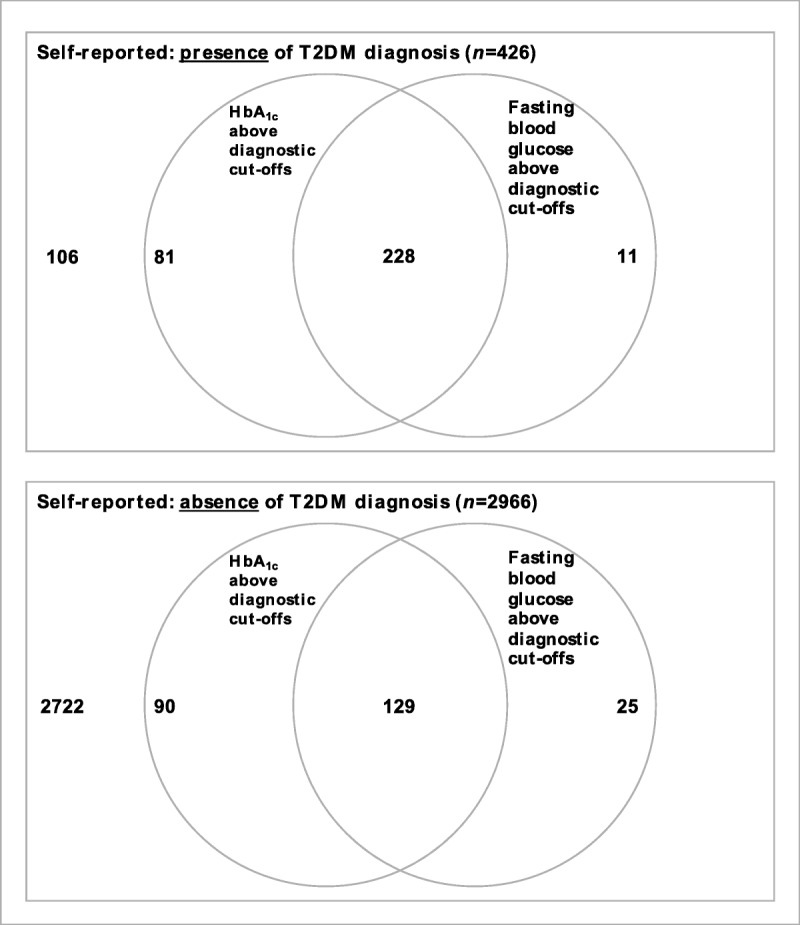
Venn diagram of participants with self-reported presence/absence of T2DM and for whom biological samples were available. T2DM = type 2 diabetes; HbA_1c_ = glycated hemoglobin;

The recommended diagnostic cutoff values for T2DM using HbA_1c_ are ≥48 mmol/mol as per IFCC and 6.5% as per DCTT, and for fasting plasma glucose, the value is ≥7.0 mmol/L (34). Among individuals with self-reported T2DM and for whom biological samples were available (*n* = 426), 320 (75.1%) had either HbA_1c_ or fasting plasma glucose, or both, above diagnostic cutoffs (Figure 1). Both HbA_1c_ and fasting plasma glucose can be within the reference range among people with well-controlled T2DM and therefore do not exclude T2DM being present. Among individuals who did not report having a T2DM diagnosis and for whom biological samples were available (*n* = 2966), 244 (8.2%) had either HbA_1c_ or fasting plasma glucose, or both, above diagnostic cutoffs (Figure 1). This suggests that there might be a small proportion of individuals in CoTaSS-2 who fulfill the diagnostic criteria for T2DM but are unaware of the disease process.

The mean (SD) BDI depression score was 4.9 (6.2; Cronbach α = .87), with 355 individuals (9.2%) scoring higher than 13, the cutoff for a clinical diagnosis of depression using the BDI. The BDI scores were positively skewed on visual inspection, with a kurtosis of 6.98, which reduced to −0.17 after log transformation of the age- and sex-regressed scores. In male individuals, the phenotypic correlations between depression and (i) self-reported T2DM diagnosis, (ii) fasting blood glucose, and (iii) HbA_1c_ were 0.06 (95% confidence interval = −0.02 to 0.14), 0.06 (0–0.11), and 0.06 (0.01–0.11), respectively. In female individuals, they were 0.15 (0.08–0.21), 0.05 (0.01–0.10), and 0.06 (0.01–0.10). Correlations stratified by zygosity and sex are summarized in Table 2. Given the small phenotypic correlation between depression and both fasting blood glucose and HbA_1c_, we focused on the genetic model-fitting between depression and self-reported diagnosis of T2DM.

**TABLE 2:**
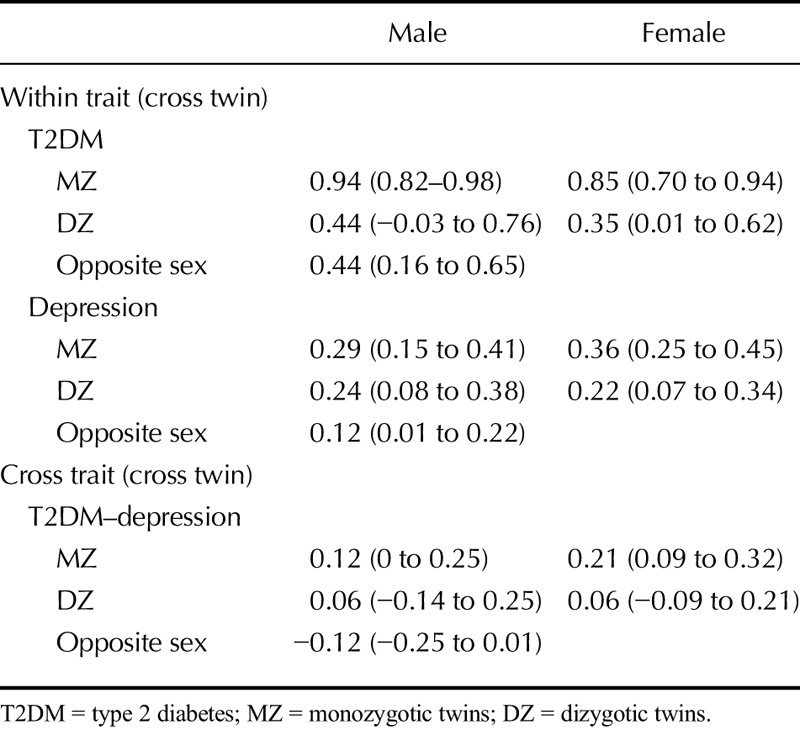
Correlations for (i) T2DM, (ii) Depression, and (iii) T2DM-Depression by Zygosity and Sex

### Genetic Model Fitting

**FIGURE 2:**
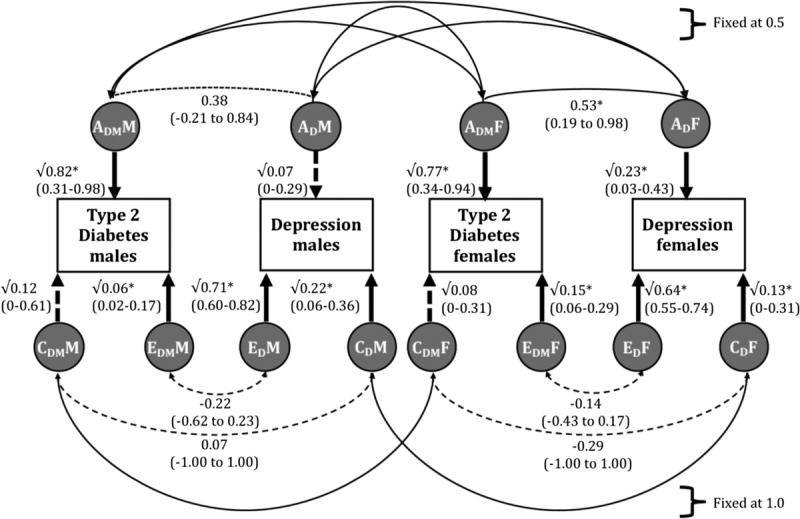
Parameter estimates of the bivariate ACE twin model for type 2 diabetes and depression. The diagram above is for best-fit sex-limitation bivariate ACE model for opposite-sex twin pairs. Factor notation: A indicates addictive genetic effects; C common environmental effects; and E unique environmental effects; subscript DM indicates type 2 diabetes and D indicates depression; M stands for male and F for female. Asterisks indicate a significant pathway.

First, a sex-limitation ACE model including quantitative and qualitative genetic sex differences was fitted (HetACEg: −2LL = 15,429.91; *df* = 7799; AIC = −168.09). Significance of qualitative genetic sex differences was tested by comparing this model with the one in which the correlation between the A and C factors across male and female individuals in opposite-sex pairs were constrained to correlate at 0.50 and 1, respectively, as is the case in same-sex DZ pairs (HetACE: −2LL = 15,432.42; *df* = 7803; AIC = −174.58). This resulted in a nonsignificant decline in model-fit (HetACEg versus HetACE: χ^2^_(*df* = 4)_ = 1.52; *p* = .82), indicating that qualitative sex differences for the genetic factors were negligible. Second, a sex-limitation ACE model including quantitative and qualitative common environmental sex differences was fitted (HetACEc: −2LL = 15,431.33; *df* = 7799; AIC = −166.67). Compared with the HetACE model, this model also showed a nonsignificant decline in fit (HetACEc versus HetACE: χ^2^_(*df* = 4)_ = 0.097; *p* > .99). Third, we tested for quantitative sex differences by equating the A, C, and E parameters across male and female individuals (HomoACE: −2LL = 15,583.57; *df* = 7812; AIC = −40.43). Compared with the HetACE model, this resulted in a significant decline in fit (HomoACEc versus HetACE: χ^2^_(*df* = 9)_ = 152.15; *p* < .0001), indicating some importance of quantitative sex differences. The best-fitting sex-limitation model with quantitative sex differences only is represented in Figure 2.

Estimates of the standardized additive genetic (heritability; a^2^), common (c^2^), and unique environment (e^2^) variance of the traits were similar across sexes for T2DM: 82% (31%–98%), 12% (0%–61%), and 6% (2%–17%), respectively in male individuals, and 77% (34%–94%), 8% (0%–31%), and 15% (6%–29%), respectively, in female individuals. For depression, the estimates are different across sex for depression: 7% (0%–29%), 22% (6%–36%), and 71% (60%–82%), respectively, in male individuals, and 23% (3%–43%), 13% (0%–31%), and 64% (55%–74%), respectively, in female individuals. The genetic correlation between T2DM and depression was nonsignificant in male individuals (0.38 [−0.21 to 0.84]) but significant in female individuals (0.53 [0.19 to 0.98]). The significant phenotypic correlation in female individuals is mainly due to correlated genetic factors.

## DISCUSSION

To our knowledge, this is the first study reporting a significant genetic overlap between T2DM and depression in female individuals in a non-Western population. Our findings in female individuals are consistent with one previous report in two large Scandinavian populations (13), with most of the phenotypic overlap observed being due to correlated genetic factors. Although the magnitude of our genetic correlation is substantially higher, the wider confidence interval (0.53 [0.19–0.98]) overlaps with estimates derived from the Swedish (0.23 [0.07–0.38]) and Danish (0.18 [0.06–0.31]) twin samples. Our findings differ from two other twin studies that did not report a genetic overlap between T2DM and depression, but the effect of sex was not explored in these two studies (11,12).

The major differences between our findings and previous reports in Western populations are observed for male individuals: first, the phenotypic correlation was nonsignificant for male individuals in our sample (0.06 [−0.02 to 0.14]), whereas it is significant in both the Swedish (male: 0.13 [0.08–0.14], female: 0.16 [0.12–0.17]) and Danish (male: 0.16 [0.12–0.20], female: 0.15 [0.12–0.20]) twin samples. Second, our best-fitting model includes the effects of common environment, whereas previous twin studies do not. This might, in part, be explained by common environmental factors being more important in explaining individual differences in depression for male individuals in non-Western populations like Sri Lanka. The significant effects of common environment factors on depression have been previously reported in a Korean twin sample of adolescents and young adult men (32%, male) (23).

Our heritability estimates for depression in males were also significantly lower than those reported in a meta-analysis (~37%) (35). A possible explanation is that there is more room for environmental factors to explain individual differences in non-Western populations, leading to a lower heritability estimate. Previous studies in the Sri Lankan population have identified male-specific environmental factors to play a role in depression, namely, unemployment, low levels of standard of living, and living in more heavily urbanized areas (36). Our finding of significant common environmental effects in male individuals might therefore reflect the differential economic and social pressures between the sexes in non-Western populations and between Western and non-Western populations in general, and their subsequent effect on developing depression.

In addition to the explanation above, it is possible that a different phenotype of depression might be captured when a questionnaire developed in a Western context is used in a non-Western population. A previous study using the Composite International Diagnostic Interview has demonstrated that the total number of depressive symptoms and pattern of symptoms endorsed were similar between the Sri Lankan and Western populations (36), suggesting some degrees of phenotypic overlap. A study in the United Kingdom has reported that people of South Asian origin were more likely to disclose somatic rather than psychological symptoms when screening for nonpsychotic psychiatric disorders, although the extent of cultural variation in expressing psychological distress remains unclear and controversial (37). Given that no specific measure of environmental factors was included in our analysis, we are merely speculating on the nature and type of environmental factors that might contribute to the T2DM-depression association in non-Western populations. If our findings are replicated in a larger non-Western population twin sample, future studies could examine whether male-specific environmental factors modulate the etiology of depression and its association with T2DM in non-Western populations. It should, however, be noted that non-Western populations are not a uniform entity, highlighting the need to widen the current evidence base of research in non-Western populations. Study-specific differences also need to be taken into account in interpreting our findings: the CoTaSS-2 sample differs from previous twin studies in the assessment of T2DM and depression (self-report questionnaire/diagnosis versus clinical diagnosis derived from hospital records/registries) and being a younger cohort.

### Limitation

A major limitation of the study is the reliance on self-report questionnaires for assessing T2DM and depression. Information on diabetes management was unavailable at the time of analysis, limiting the scope of cross-checking self-reported items. We did explore the use of HbA_1c_ as a proxy marker for T2DM, but the phenotypic correlation with depression was very small in magnitude. HbA_1c_ is a useful clinical biomarker for assessing glycemic status and guiding treatment decisions for people with T2DM. It can, however, be within the reference range among people with well-controlled T2DM. For example, ~25% of individuals with T2DM in our study have an HbA_1c_ level of <48 mmol/mol (6.5%), the recommended cutoff for diagnosing T2DM. HbA_1c_ alone might therefore not be a sufficiently reliable tool for recognizing T2DM, especially during the early stages of the disease. In addition, being diagnosed as having T2DM, and initiating and implementing the associated diabetes self-management might have a greater impact on the development of depression than HbA_1c_ alone, explaining the differential phenotypic correlations between depression and (i) T2DM diagnosis and (ii) HbA_1c_.

For depression, the BDI captures depressive symptoms for the past 2 weeks and is not aimed to establish a diagnosis of major depressive disorder. The main rationale of selecting BDI as an outcome measure for depression in this study is its high internal consistency in both psychiatric and nonpsychiatric samples, rendering it appropriate for CoTaSS-2, a population-based cohort (38,39). It has also demonstrated high convergent validity with other rating scales for depression and discriminated reliably between individual with and without depressive symptoms (38,39). Psychiatric disorders remain underrecognized in Sri Lanka. A scarcity of mental health resources and stigma have been identified as major barriers for communities to seek care (40). A recent national survey of self-reported health in Sri Lanka reported that only 23% of individuals reporting to have a mental illness receive any treatment (41). In addition, the self-report nature of a questionnaire can affect its results owing to social desirability and respondent educational attainment (42). Thus, a more comprehensive approach would be to conduct a structured diagnostic interview to screen for mental illnesses in the CoTASS-2 sample, but it is both time- and labor-intensive.

Information on antidepressant was also not available at the time of analysis, and thus, it is possible that individuals who were actively depressed and receiving antidepressant treatment were included in the COTASS-2 sample. This can potentially affect their responses on the BDI. To further complicate the picture, mixed results have previously been reported between the association between antidepressants and glycemic control. For example, a cross-sectional study using a large representative population of US adults without a diagnosis of diabetes (*n* = 6141) concluded that antidepressant use was not associated with an increased risk for abnormalities in glycemic control or undetected diabetes (43). A longitudinal study in adults who were at high risk for developing T2DM, however, demonstrated that antidepressant use is associated with elevated inflammatory markers and incident T2DM (*n* = 3187) (44). The association between antidepressants use and T2DM has not been extensively examined in non-Western populations, and thus, the effect of antidepressants on HbA_1c_ remains uncertain in the Sri Lankan population.

Adopting a multi-informant approach, such as using a valid and reliable diagnostic interview for depression or cross-validating our measures with a clinical diagnosis and medication registry, could potentially strengthen our finding. Our study also uses cross-sectional data, and thus, we cannot determine the extent by which individuals later develop depression or T2DM after being recruited into the study. A longitudinal design will allow us to examine changes in genetic and environmental influences in the clinical course of T2DM. Lastly, limitations of the classical twin model apply, namely, the equal environment assumption and the assumption of negligible correlations between the A, C, and E factors (45). In addition, it is important to note that the heritability estimates derived from GWAS using techniques such as genome-wide complex trait analysis and linkage disequilibrium score regression are generally half when compared with twin studies (46). The discrepancy currently remains unclear and can in part be due to GWAS only capturing the effects of single nucleotide polymorphisms with a minor allele frequency of greater than 1%. Genome-wide complex trait analysis also does not include nonadditive interactions, such as gene-gene or gene-environment. At this stage, we have only begun to uncover the complex genetic underpinning of the T2DM-depression association.

## CONCLUSIONS

Our study strengthens previous reports of genetic factors playing an important role in the mechanism underlying the T2DM-depression link in female individuals by replicating the finding in a non-Western population and thus demonstrating the generalizability of the finding. The reason for the discrepancy in findings between twin and GWAS studies is currently unclear, and it seems that we have only begun to uncover the complex genetic underpinning of the association between T2DM and depression.

## References

[1] Rotella F Mannucci E Diabetes mellitus as a risk factor for depression. A meta-analysis of longitudinal studies. Diabetes Res Clin Pract 2013;99:98–104. 23265924 10.1016/j.diabres.2012.11.022

[2] Mezuk B Eaton WW Albrecht S Golden SH Depression and type 2 diabetes over the lifespan: a meta-analysis. Diabetes Care 2008;31:2383–90. 19033418 10.2337/dc08-0985 PMC2584200

[3] Lustman PJ Anderson RJ Freedland KE de Groot M Carney RM Clouse RE Depression and poor glycemic control: a meta-analytic review of the literature. Diabetes Care 2000;23:934–42. 10895843 10.2337/diacare.23.7.934

[4] De Groot M Anderson R Freedland KE Clouse RE Lustman PJ Association of depression and diabetes complications: a meta-analysis. Psychosom Med 2001;63:619–30. 11485116 10.1097/00006842-200107000-00015

[5] Katon WJ Rutter C Simon G Lin EH Ludman E Ciechanowski P Kinder L Young B Von Korff M The association of comorbid depression with mortality in patients with type 2 diabetes. Diabetes Care 2005;28:2668–72. 16249537 10.2337/diacare.28.11.2668

[6] Renn BN Feliciano L Segal DL The bidirectional relationship of depression and diabetes: a systematic review. Clin Psychol Rev 2011;31:1239–46. 21963669 10.1016/j.cpr.2011.08.001

[7] Kan C Silva N Golden SHH Rajala U Timonen M Stahl D Ismail K A systematic review and meta-analysis of the association between depression and insulin resistance. Diabetes Care 2013;36:480–9. 23349152 10.2337/dc12-1442 PMC3554272

[8] Laake JP Stahl D Amiel SA Petrak F Sherwood RA Pickup JC Ismail K The association between depressive symptoms and systemic inflammation in people with type 2 diabetes: findings from the South London Diabetes Study. Diabetes Care 2014;37:2186–92. 24842983 10.2337/dc13-2522

[9] Doyle T Halaris A Rao M Shared neurobiological pathways between type 2 diabetes and depressive symptoms: a review of morphological and neurocognitive findings. Curr Diab Rep 2014;14:560. 25381209 10.1007/s11892-014-0560-7

[10] Moulton CD Pickup JC Ismail K The link between depression and diabetes: the search for shared mechanisms. Lancet Diabetes Endocrinol 2015;3:461–71. 25995124 10.1016/S2213-8587(15)00134-5

[11] Scherrer JF Xian H Lustman PJ Franz CE McCaffery J Lyons MJ Jacobson KC Kremen WS A test for common genetic and environmental vulnerability to depression and diabetes. Twin Res Hum Genet 2011;14:169–72. 21425899 10.1375/twin.14.2.169 PMC3480187

[12] Mezuk B Heh V Prom-Wormley E Kendler KS Pedersen NL Association between major depression and type 2 diabetes in midlife: findings from the Screening Across the Lifespan Twin Study. Psychosom Med 2015;77:559–66. 25967355 10.1097/PSY.0000000000000182 PMC4459909

[13] Kan C Pedersen NLL Christensen K Bornstein SRR Licinio J MacCabe JHH Ismail K Rijsdijk F Genetic overlap between type 2 diabetes and depression in Swedish and Danish twin registries. Mol Psychiatry 2016;21:903–9. 27021822 10.1038/mp.2016.28 PMC5414070

[14] Samaan Z Garasia S Gerstein HC Engert JC Mohan V Diaz R Anand SS Meyre D Lack of association between type 2 diabetes and major depression: epidemiologic and genetic evidence in a multiethnic population. Transl Psychiatry 2015;5:e618. 26261886 10.1038/tp.2015.113 PMC4564566

[15] Clarke TK Obsteter J Hall LS Hayward C Thomson PA Smith BH Padmanabhan S Hocking LJ Deary IJ Porteous DJ McIntosh AM Investigating shared aetiology between type 2 diabetes and major depressive disorder in a population based cohort. Am J Med Genet B Neuropsychiatr Genet 2017;174:227–34. 27480393 10.1002/ajmg.b.32478 PMC5363226

[16] Bulik-Sullivan B Finucane HK Anttila V Gusev A Day FR Loh PR Duncan L Perry JR Patterson N Robinson EB Daly MJ Price AL Neale BM An atlas of genetic correlations across human diseases and traits. Nat Genet 2015;47:1236–41. 26414676 10.1038/ng.3406 PMC4797329

[17] Haljas K Amare AT Alizadeh BZ Hsu Y-H Mosley T Newman A Murabito J Tiemeier H Tanaka T van Duijn C Ding J Llewellyn DJ Bennett DA Terracciano A Launer L Ladwig K-H Cornelis MC Teumer A Grabe H Kardia SLR Ware EB Smith JA Snieder H Eriksson JG Groop L Räikkönen K Lahti J Bivariate genome-wide association study of depressive symptoms with type 2 diabetes and quantitative glycemic traits. Psychosom Med 2018;80:242–51. 29280852 10.1097/PSY.0000000000000555 PMC6051528

[18] Hashim NA Ariaratnam S Salleh MR Said MA Sulaiman AH Depression and associated factors in patients with type 2 diabetes mellitus. East Asian Arch Psychiatry 2016;26:77–82. 27377489

[19] Arshad AR Alvi KY Frequency of depression in type 2 diabetes mellitus and an analysis of predictive factors. J Pak Med Assoc 2016;66:425–9. 27122269

[20] Park CY Kim SY Gil JW Park MH Park JH Kim Y Depression among Korean adults with type 2 diabetes mellitus: Ansan-community-based epidemiological study. Osong Public Health Res Perspect 2015;6:224–32. 26473089 10.1016/j.phrp.2015.05.004 PMC4588455

[21] Lim GY Tam WW Lu Y Ho CS Zhang MW Ho RC Prevalence of depression in the community from 30 countries between 1994 and 2014. Sci Rep 2018;8:2861. 29434331 10.1038/s41598-018-21243-x PMC5809481

[22] Ramachandran A Ma RC Snehalatha C Diabetes in Asia. Lancet 2010;375:408–18. 19875164 10.1016/S0140-6736(09)60937-5

[23] Siribaddana SH Ball HA Hewage SN Glozier N Kovas Y Dayaratne D Sumathipala A McGuffin P Hotopf M Colombo Twin and Singleton Study (CoTASS): a description of a population based twin study of mental disorders in Sri Lanka. BMC Psychiatry 2008;8:49. 18588676 10.1186/1471-244X-8-49 PMC2475532

[24] Alonso-Moran E Satylganova A Orueta J Nuno-Solinis R Prevalence of depression in adults with type 2 diabetes in the Basque Country: relationship with glycaemic control and health care costs. BMC Public Health 2014;14:769. 25073532 10.1186/1471-2458-14-769 PMC4129099

[25] Anderson RJ Freedland KE Clouse RE Lustman PJ The prevalence of comorbid depression in adults with diabetes: a meta-analysis. Diabetes Care 2001;24:1069–78. 11375373 10.2337/diacare.24.6.1069

[26] Ali S Stone MA Peters JL Davies MJ Khunti K The prevalence of co-morbid depression in adults with type 2 diabetes: a systematic review and meta-analysis. Diabet Med 2006;23:1165–73. 17054590 10.1111/j.1464-5491.2006.01943.x

[27] Jayaweera K Aschan L Pannala G Adikari A Glozier N Ismail K Pariante CM Rijsdijk F Siribaddana S Zavos HMS Zunszain PA Sumathipala A Hotopf M The Colombo Twin and Singleton Follow-up Study: a population based twin study of psychiatric disorders and metabolic syndrome in Sri Lanka. BMC Public Health 2018;18:145. 29343229 10.1186/s12889-017-4992-2 PMC5773033

[28] Beck AT Steer RA Brown GK Manual for the Beck Depression Inventory-II. San Antonio, TX: Psychological Corporation; 1996.

[29] Sumathipala A Murray J New approach to translating instruments for cross-cultural research: a combined qualitative and quantitative approach for translation and consensus generation. Int J Methods Psychiatr Res 2000;9:87–95.

[30] Ooki S Yamada K Asaka A Zygosity diagnosis of twins by questionnaire for twins’ mothers. Acta Genet Med Gemellol (Roma) 1993;42:17–22. 8191857 10.1017/s0515283600042244

[31] Neale MC Cardon LR Methodology for Genetic Studies of Twins and Families in Neurogenetics. The Netherlands: Kluwer Academic Publishers; 1992.

[32] Boker S Neale M Maes H Wilde M Spiegel M Brick T Spies J Estabrook R Kenny S Bates T Mehta P Fox J OpenMx: an open source extended structural equation modeling framework. Psychometrika 2011;76:306–17. 23258944 10.1007/s11336-010-9200-6 PMC3525063

[33] Akaike H Factor analysis and AIC. Psychometrika 1987;52:317–32.

[34] World Health Organization. Definition and Diagnosis of Diabetes Mellitus and Intermediate Hyperglycemia: Report of a WHO/IDF Consultation. Geneva; 2006.

[35] Sullivan PF Neale MC Kendler KS Genetic epidemiology of major depression: review and meta-analysis. Am J Psychiatry 2000;157:1552–62. 11007705 10.1176/appi.ajp.157.10.1552

[36] Ball HA Siribaddana SH Kovas Y Glozier N McGuffin P Sumathipala A Hotopf M Epidemiology and symptomatology of depression in Sri Lanka: a cross-sectional population-based survey in Colombo District. J Affect Disord 2010;123:188–96. 19762085 10.1016/j.jad.2009.08.014 PMC2946561

[37] Hussain F Cochrane R Depression in south Asian women living in the UK: a review of the literature with implications for service provision. Transcult Psychiatry 2004;41:253–70. 15446723 10.1177/1363461504043567

[38] Richter P Werner J Heerlein A Kraus A Sauer H On the validity of the Beck Depression Inventory. Psychopathology 1998;31:160–8. 9636945 10.1159/000066239

[39] Wang YP Gorenstein C Psychometric properties of the Beck Depression Inventory-II: a comprehensive review. Braz J Psychiatry 2013;35:416–31. 24402217 10.1590/1516-4446-2012-1048

[40] D’Souza R Singh B The mental health challenge in Sri Lanka from working within the disaster area. World Psychiatry 2005;4:68. 16633510 PMC1414733

[41] Ministry of National Policies and Economic Affairs. National Survey of self reported health in Sri Lanka 2014. Available at: http://www.statistics.gov.lk/social/National Survey on Self-reported Health-2014.pdf. January 7, 2020.

[42] Cronbach LJ Essentials of Psychological Testing. Harper & Row; 1990 Available from: https://books.google.co.uk/books?id=AwQlAQAAIAAJ.

[43] Mojtabai R Antidepressant use and glycemic control. Psychopharmacology 2013;227:467–77. 23334176 10.1007/s00213-013-2972-5

[44] de Groot M Marrero D Mele L Doyle T Schwartz F Mather KJ Goldberg R Price DW Ma Y Knowler WC Depressive symptoms, antidepressant medication use, and inflammatory markers in the diabetes prevention program. Psychosom Med 2018;80:167–73. 29016549 10.1097/PSY.0000000000000535 PMC5794527

[45] Golan D Lander ES Rosset S Measuring missing heritability: inferring the contribution of common variants. Proc Natl Acad Sci U S A 2014;111:E5272–81. 25422463 10.1073/pnas.1419064111 PMC4267399

[46] Plomin R Simpson MA The future of genomics for developmentalists. Dev Psychopathol 2013;25:1263–78. 24342839 10.1017/S0954579413000606 PMC3967388

